# Lack of clinical efficacy of imatinib in metastatic melanoma

**DOI:** 10.1038/sj.bjc.6602529

**Published:** 2005-04-20

**Authors:** S Ugurel, R Hildenbrand, A Zimpfer, P La Rosée, P Paschka, A Sucker, P Keikavoussi, J C Becker, W Rittgen, A Hochhaus, D Schadendorf

**Affiliations:** 1 Skin Cancer Unit, German Cancer Research Center Heidelberg and Department of Dermatology, University Hospital of Mannheim, Theodor-Kutzer-Ufer 1, D-68167 Mannheim, Germany; 2 Department of Pathology, University Hospital of Mannheim, 68167 Mannheim, Germany; 3 III. Medizinische Klinik, Fakultät für Klinische Medizin Mannheim der Universität Heidelberg, 68167 Mannheim, Germany; 4 Department of Dermatology, University Hospital of Würzburg, 97080 Würzburg, Germany; 5 Central Unit of Biostatistics, German Cancer Research Center, 69120 Heidelberg, Germany

**Keywords:** imatinib, melanoma, c-kit, PDGF-R

## Abstract

This two-centre phase-II trial aimed at investigating the efficacy of imatinib in metastasised melanoma patients in correlation to the tumour expression profile of the imatinib targets c-kit and platelet-derived growth factor receptor (PDGF-R). The primary study end point was objective response according to RECIST, secondary end points were safety, overall and progression-free survival. In all, 18 patients with treatment-refractory advanced melanoma received imatinib 800 mg day^−1^. In 16 evaluable patients no objective responses could be observed. The median overall survival was 3.9 months, the median time to progression was 1.9 months. Tumour biopsy specimens were obtained from 12 patients prior to imatinib therapy and analysed for c-kit, PDGF-R*α* and -R*β* expression by immunohistochemistry. In four cases, cell lines established from these tumour specimens were tested for the antiproliferative effects of imatinib and for functional mutations of genes encoding the imatinib target molecules. The tumour specimens stained positive for CD117/c-kit in nine out of 12 cases (75%), for PDGF-R*α* in seven out of 12 cases (58%) and for PDGF-R*β* in eight out of 12 cases (67%). The melanoma cell lines showed a heterogenous expression of the imatinib target molecules without functional mutations in the corresponding amino-acid sequences. *In vitro* imatinib treatment of the cell lines showed no antiproliferative effect. In conclusion, this study did not reveal an efficacy of imatinib in advanced metastatic melanoma, regardless of the expression pattern of the imatinib target molecules c-kit and PDGF-R.

At present, no treatment options are available for melanoma patients with advanced metastastic disease that provide either sufficient response rates or a significant prolongation of overall survival (Eigentler *et al*, 2003; Ugurel and Schadendorf, 2003). Thus, despite all therapeutic efforts, the 5-year-survival rate in melanoma patients with metastases to the subcutis and distant lymph nodes is less than 20% (Balch *et al*, 2001), in patients with metastases to the lung and other organs it ranges between 6.7 and 9.5% (Balch *et al*, 2001). Chemotherapy with dacarbacine (DTIC) does actually apply as the standard treatment regimen in advanced melanoma, leading to response rates between 9.9 and 18% (Crosby *et al*, 2001). Polychemotherapy as well as therapy regimens combining cytostatics with cytokines do not add any substantial survival benefit to DTIC monotherapy (Crosby *et al*, 2001; Eigentler *et al*, 2003).

Due to this unfavourable situation, recent approaches to gain a stronger efficacy against melanoma focused on new target-directed therapeutic strategies. Here, in particular, signalling pathways and their key molecules, such as bcl-2 (Jansen *et al*, 2000) or Ras-Raf-MAPK (Collisson *et al*, 2003; Sumimoto *et al*, 2004), became targets of interest. A group of signal transductory molecules of crucial importance for the regulation of cell growth and survival are the receptor tyrosine kinases. A small therapeutic molecule designed to selectively inhibit these kinases is imatinib mesylate (Glivec®, formerly STI571, Novartis, Basel, Switzerland). Imatinib selectively binds to the tyrosine kinases Abl and Kit, as well as the platelet-derived growth factor receptors (PDGF-R) alpha and beta. Due to its mechanisms of action, imatinib has been used successfully in chronic myelogenous leukaemia (CML) (Druker *et al*, 2001) and in gastrointestinal stromal tumours (GIST) (van Oosterom *et al*, 2001).

Melanoma cells are known to express c-kit, PDGF-R and Abl (Worm *et al*, 1993; Montone *et al*, 1997; Welker *et al*, 2000; Potti *et al*, 2003; Shen *et al*, 2003). Moreover, based on experimental *in vitro* data gained from melanoma cell lines, autocrine growth loop mechanisms were postulated for the receptor–ligand interaction of PDGF-R and PDGF, as well as of Kit and stem cell factor (SCF) (Harsh *et al*, 1990; DiPaola *et al*, 1997). Thus, by inhibiting cell viability and proliferation via inhibition of receptor tyrosine kinases, imatinib might be a successful therapeutic in malignant melanoma. A first study investigating the growth behaviour of two human melanoma cell lines in athymic nude mice showed that imatinib treatment of these mice resulted in an inhibition of PDGF-R phosphorylation but no decrease in tumour size (McGary *et al*, 2004). In contrast, a recent report on murine B16F10 melanoma cells injected into C57B16 mice revealed a strong and efficient tumour growth inhibition by imatinib (Redondo *et al*, 2004). The same study also demonstrated a growth inhibition effect of imatinib on B16F10 melanoma cells *in vitro*.

The present study was designed to investigate the safety and efficacy of imatinib in patients with advanced metastatic melanoma in correlation to the tumour expression profile of its target molecules c-kit and PDGF-R.

## PATIENTS AND METHODS

### Study design

This study was designed as a two-centre prospective open-label phase-II trial to investigate the safety and efficacy of imatinib in treatment-refractory metastatic malignant melanoma. The primary study end point was objective response, secondary end points were overall survival, time to progression and toxicity. All end points were evaluated on an intention-to-treat (ITT) and on a per protocol (PP) basis. Toxicity was analysed in all patients who received study treatment.

### Patient population

Patients with histologically confirmed metastastic melanoma were enrolled into the study in accordance with the following eligibility criteria: melanoma disease refractory to at least one previous treatment regimen; at least one measurable target lesion following the response evaluation criteria in solid tumours (RECIST) (Therasse *et al*, 2000); no brain metastases; ECOG performance status ⩽2; age above 18 years; adequate bone marrow function (leukocytes ⩾3000 *μ*l^−1^, platelets ⩾100 000 *μ*l^−1^); and satisfactory hepatic and renal functions. Patients with severe and/or uncontrolled medical diseases other than melanoma were excluded from the study. The study protocol was approved by the Institutional Review Boards of the participating centres and a written informed consent was signed by all patients prior to enrolment.

### Cells and tissues

Biopsy specimens from metastatic lesions were taken from 12 patients prior to the onset of study treatment with written informed consent. Specimens were divided into two parts, with one being subsequently embedded into paraffin and the other used for cell culture. For cell culture purposes, the biopsy specimens were minced and thereafter maintained in RPMI 1640 (Life Technologies, Grand Island, NY, USA) supplemented with 10% foetal calf serum (Life Technologies), 5 mM L-glutamine, 100 U ml^−1^ penicillin and 100 *μ*g ml^−1^ streptomycin at 37°C in a humidified 5% CO_2_ atmosphere. Established cell lines were used for further investigations no earlier as after six to eight passages.

### Immunohistochemical analysis

Formalin-fixed paraffin-embedded 5 *μ*m tissue sections were pretreated with 0.1% protease type XIV (P-5147, Sigma-Aldrich, Munich, Germany) for 10 min at 37°C prior to the incubation with primary antibodies. The following primary antibodies were used: anti-CD117/c-kit (rabbit polyclonal IgG A4502, DakoCytomation, Glostrup, Denmark), anti-PDGF-R*α* (rabbit polyclonal IgG #07-276, Upstate, Lake Placid, NY, USA) and anti-PDGF-R*β* (rabbit polyclonal IgG #06-498, Upstate). The tissue sections were incubated with the primary antibodies diluted 1 : 100 for 1 h at room temperature. After several washes, the bound primary antibodies were visualised using streptavidin–biotin labelling (LSAB-kit, DakoCytomation) according to the manufacturer's instructions. The resulting immunostaining appears in red colour; nuclei were counterstained with haematoxylin (blue colour). In each case, negative controls were performed by substituting the primary antibodies by irrelevant IgG antibodies. The intensity of immunostaining was graded as negative (−), weak (+), moderate (++) or strong (+++). Melanomas with weak to strong staining in more than 10% of the tumour cells were considered as positive.

The melanoma cell lines were allowed to grow to confluence. After gentle detachment by incubation with 0.05% EDTA/PBS for 10 min, the cells were centrifuged at 1500 **g** for 5 min. The obtained cytospin preparations were fixed with 2% paraformaldehyde, air-dried, and subsequently pretreated with 0.1% protease type XIV for 10 min at 37°C. Thereafter, the specimens were incubated with the above-mentioned primary antibodies at a concentration of 1 : 100 overnight (ckit and PDGF-R*α*) and for 1 h (PDGF-R*β*) at room temperature. After several washes, the bound primary antibodies were visualised using streptavidin–biotin labelling. The resulting immunostaining appears in red colour; nuclei were counterstained in blue with haematoxylin. Negative controls were performed by substituting the primary antibodies by irrelevant IgG antibodies. The intensity of immunostaining was graded as negative (−), weak (+), and moderate to strong (++).

### Mutation analysis

RNA from melanoma cell lines was extracted by CsCl gradient centrifugation as described previously (Cross *et al*, 1993). RNA was transcribed into copy DNA (cDNA) using random hexamer primers and Moloney murine leukaemia virus reverse transcriptase. The sequences of the primers used for PCR amplification of the entire c-kit coding region are published elsewhere (Longley *et al*, 1999). In all, 3 *μ*l of cDNA was amplified for 31 cycles of 1 min at 94°C, 1 min at 58°C and 1 min at 72°C in 50 *μ*l containing 1 *μ*M primers, 1.8 mM MgCl_2_, 0.2 mM each of all four dNTPs, 20 mM Tris-HCl (pH 8.4), 50 mM KCl and 1.5 U of *Taq* polymerase. Specific primers were designed to amplify the transmembrane, juxtamembrane and kinase regions of PDGF-R*α* and -R*β* (Table 1). The PCRs were performed in a final volume of 25 *μ*l containing 0.8 *μ*M primers, 1.8 mM MgCl_2_, 0.24 mM each of all four dNTPs, 20 mM Tris-HCl (pH 8.4), 50 mM KCl, 4 *μ*l of cDNA and 1.5 U of *Taq* polymerase. The thermal profile of 35 amplification cycles was: 94°C for 50 s, 62°C for 45 s, followed by 72°C for 1 min. PCR products were visualised on 1.5% agarose gels stained with ethidium bromide. The products were sequenced and checked for mutations by comparison with reference databases (NM_000222 for c-kit, NM_006206 for PDGF-R*α*, NM_002609 for PDGF-R*β*).

### Cytotoxicity assay

To test the cytotoxic effect of imatinib on melanoma cell lines *in vitro*, MTS assays (Mosmann, 1983) were performed using the CellTiter 96 Aqueous One Solution Cell Proliferation Assay kit (Promega, Mannheim, Germany) following the manufacturer's instructions. The imatinib-sensitive, Bcr–Abl-positive CML cell line Lama84 (Deininger *et al*, 1997) was used as a control. The cells were plated into 96-well microtitre plates in quadruplicate at a concentration of 5 × 10^3^ cells well^−1^ and exposed to escalating doses of imatinib ranging from 0 to 10 *μ*M. These doses were selected according to a predescribed range of clinical relevance (Peng *et al*, 2004), with a maximum of 10 *μ*M being beyond clinical feasibility. After 72 h of incubation, the colour reaction was performed and subsequently recorded at 490 nm using an ELISA plate reader. The inhibition of cell growth under exposure with imatinib was calculated as the percentage of cell growth with medium alone.

### Treatment plan

The study treatment regimen was imatinib (Glivec®, Novartis, Basel, Switzerland) 400 mg p.o. bid (800 mg daily) for 8 weeks. Thereafter, treatment was continued at a tumour response of stable disease or better. Treatment was stopped at any time point due to disease progression or intolerable side effects. Recommended concomitant medications were metoclopramide in case of nausea and loperamide in case of diarrhoea, if required. Toxicity was evaluated using common toxicity criteria (CTC) (http://ctep.cancer.gov/reporti ng/ctc.html) and assessed at weekly intervals within the first 4 weeks of therapy, followed by 2-week intervals thereafter. Imatinib doses were adjusted due to toxicities as described previously (Demetri *et al*, 2002). After completion of study treatment, the patients were followed in at least 2 months intervals for the first 6 months and at least every 3 months afterwards.

### Response and survival assessment

Patients who completed at least 1 week of study treatment were considered evaluable for response. Tumour response was assessed by CT and/or MRI imaging in 8-week intervals and subsequently evaluated according to the RECIST guidelines (Therasse *et al*, 2000). Best response was defined as the best tumour response recorded from the start of treatment until removal of the patient from the trial. Overall survival and time to progression were measured from onset of study treatment until death or disease progression, respectively. If no such event occurred, the date of the last patient contact was used as the end point of OS or TTP assessment (censored observation).

### Statistical analysis

Patient recruitment was outlined as a total of 30 patients evaluable for objective response. A first interims analysis of imatinib efficacy was performed after the enrolment of 16 evaluable patients. Since no objective response could be achieved in this patient cohort, recruitment into the study was stopped at that time point, with a current number of 18 patients enrolled.

Survival curves were calculated and graphically presented using the Kaplan–Meier method for censored failure time data. The database was frozen on June 30, 2004. Statistical analyses were performed using the statistical packages ADAM of the Biostatistics Unit of the German Cancer Research Center and SAS for Windows Version 8.1 (SAS Institute Inc., Cary, NC, USA).

## RESULTS

### Patient characteristics and study flow

**Figure 1:**
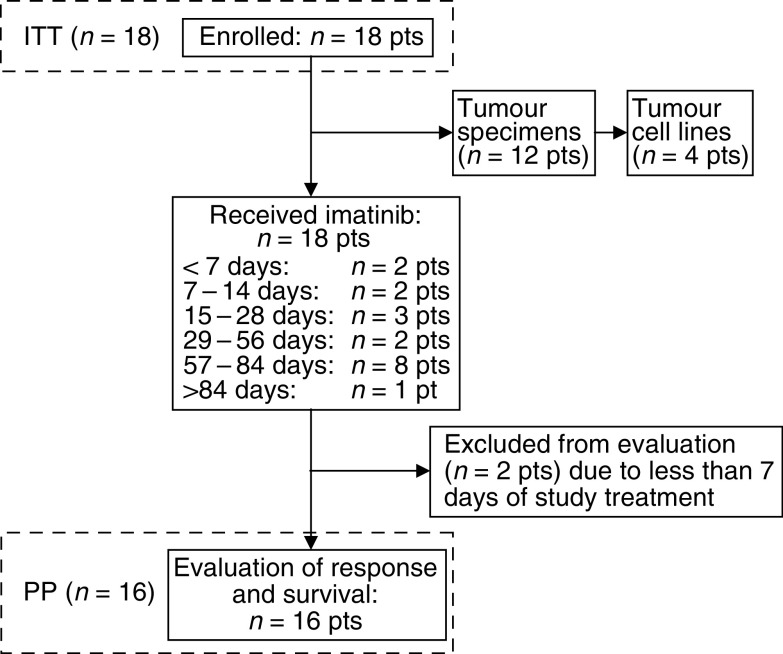
Schematic presentation of the study flow. For further details, see Table 2.

Between April 2002 and February 2003, 18 patients (ITT) with a median age of 54.2 years (range 38.9–72.0 years) were recruited into the study from two centres, Mannheim (15 patients) and Wuerzburg (three patients) (Figure 1). The prognostic indices of the study cohort at the time of enrolment were poor: 12 out of 18 patients (67%) presented with an ECOG performance score >0, 16 out of 18 (88%) were staged as AJCC M1c, and 10 out of 18 (56%) had progressed under two or more previous therapy regimens. Detailed patient characteristics are provided in Table 2.

In all, 12 biopsy specimens from metastatic lesions were obtained from 12 different study patients prior to the onset of imatinib treatment. Detailed information about the specimens’ origin is provided in Table 3. Melanoma cell lines (Ma-Mel-46, Ma-Mel-52, Ma-Mel-54a, Ma-Mel-59a) could be established from four of these specimens (Table 4).

### Immunohistochemical staining

**Figure 2:**
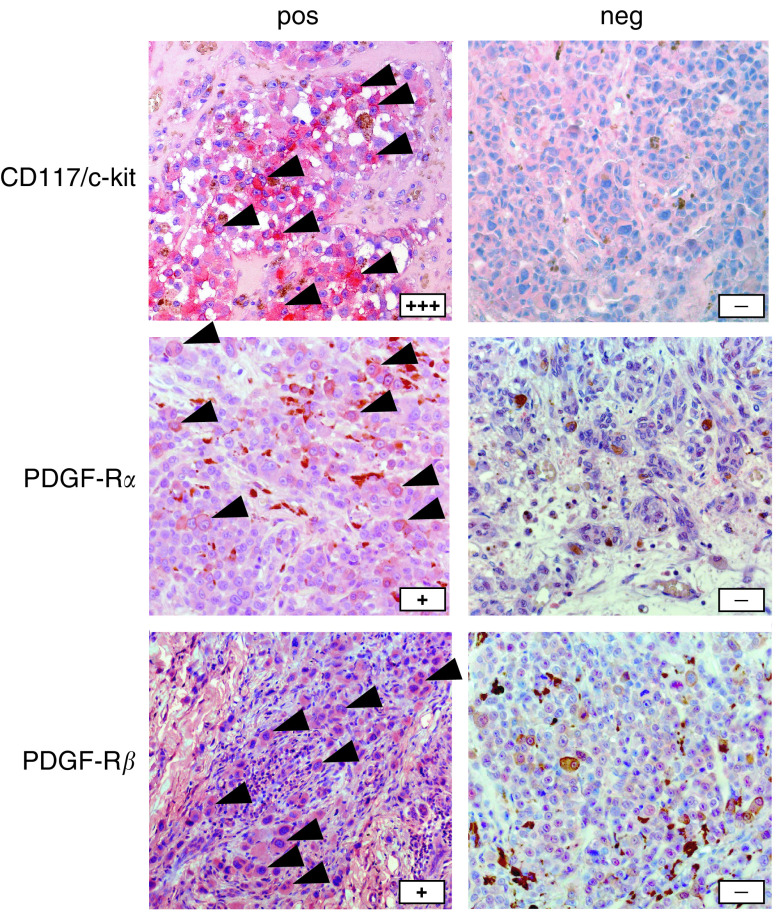
Immunohistochemical staining of CD117/c-kit, PDGF-R*α* and PDGF-R*β* in biopsy specimens of metastatic lesions obtained from six different melanoma patients prior to imatinib treatment. One representative example of each, positive and negative staining, is presented. Magnification × 100.

The paraffin-embedded sections derived from metastatic tissue specimen were reviewed by one of the authors (RH). The clinical diagnosis of melanoma metastasis could be verified in all 12 cases. Immunohistochemical staining revealed a positivity of the melanoma cells for CD117/c-kit in nine out of 12 cases (75%), for PDGF-R*α* in seven out of 12 cases (58%) and for PDGF-R*β* in eight out of 12 cases (67%). Representative cases of positive and negative staining results are provided in Figure 2. Detailed staining characteristics are given in Table 3.

**Figure 3:**
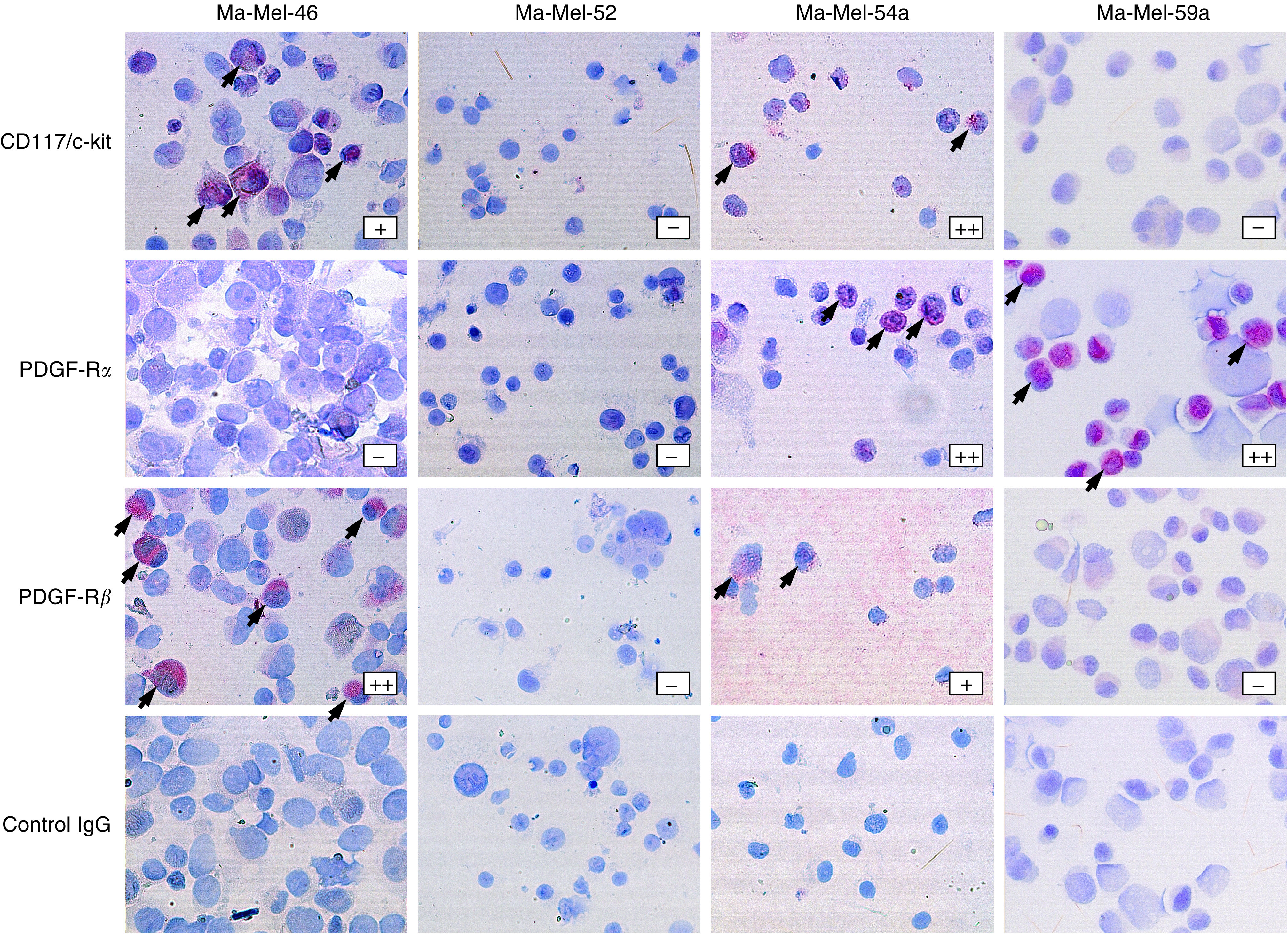
Immunohistochemical staining of CD117/c-kit, PDGF-R*α* and PDGF-R*β* in cytospin specimens of four patient-derived melanoma cell lines. Staining with irrelevant control IgG served as control. Magnification × 200.

Staining of the cytospin preparations of the four patient-derived melanoma cell lines showed two cell lines (Ma-Mel-46 and Ma-Mel-54a) expressing almost all of the three receptor tyrosine kinases tested in a weak to strong pattern (besides Ma-Mel-46 staining negative for PDGF-R*α*), whereas the other two cell lines (Ma-Mel 52 and Ma-Mel-59a) were nearly negative for those molecules (besides Ma-Mel-59a staining positive for PDGF-R*α*) (Table 4; Figure 3). Staining discrepancies were observed between the tissue samples and the corresponding cell lines (Table 4).

### Mutation analysis of c-kit, PDGF-R*α* and -R*β*

Sequencing of the entire c-kit coding region was performed successfully in three melanoma cell lines (Ma-Mel-46, Ma-Mel-52 and Ma-Mel-54a). The cell line Ma-Mel-59a showed only a weak expression of c-kit at the RNA level, not allowing a reliable sequencing of the PCR products. No mutations or base variants were found in any of the analysed c-kit sequences. In all four cell lines, the regions encoding for the transmembrane, juxtamembrane and kinase domains of PDGF-R*α* and -R*β* were analysed. The obtained nucleotide sequences revealed base changes and known polymorphisms that do not alternate the amino-acid sequence.

### *In vitro* cytotoxicity

**Figure 4:**
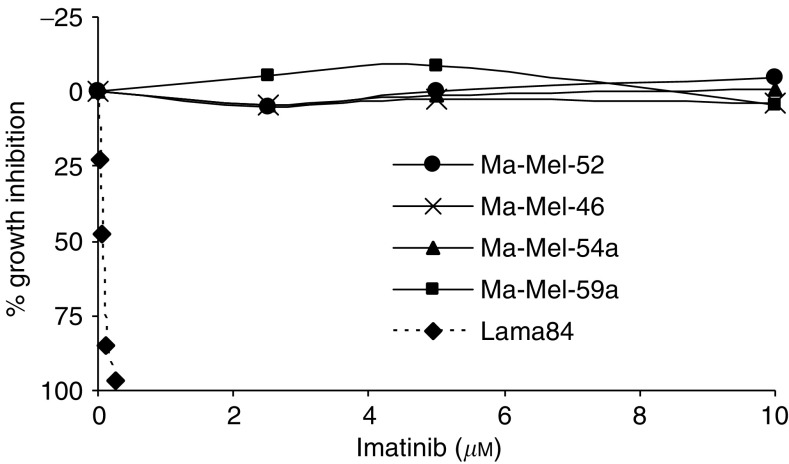
Growth inhibition by imatinib on four patient-derived melanoma cell lines (Ma-Mel-46, Ma-Mel-52, Ma-Mel-54a, Ma-Mel-59a) and one CML cell line (Lama84). Data are represented as cell growth inhibition after 72 h of imatinib exposure as percentage of growth after incubation with medium alone. The depicted data points are means generated from three independent experiments, each assayed in quadruplicates.

As shown in Figure 4, none of the four cell lines tested showed a significant growth inhibition at imatinib concentrations up to 10 *μ*M. In contrast, Bcr–Abl-positive Lama84 cells were inhibited with an IC_50_ of approximately 62.5 nM, confirming the selective activity of imatinib in Bcr–Abl-positive CML cells.

### Treatment-related toxicity

Common toxicity criteria grade 3 or 4 treatment-related toxicities, as well as all toxicities leading to a dose modification of imatinib, are summarised in Table 5. Grade 3 or 4 toxicities were experienced by five patients (27.8%); dose modifications of imatinib became necessary in seven patients (38.9%). Three patients (16.7%) discontinued study treatment due to severe adverse events (intestinal perforation, arterial thromboembolism, suicide attempt). In one case (intestinal perforation) the severe adverse event led to a fatal outcome.

### Response to treatment

All 18 patients enrolled into the study (ITT population) received study treatment; the treatment durations are presented in Figure 1. Two patients had to be excluded from the PP evaluation due to a treatment duration of less than 1 week, leading to a total number of 16 patients evaluable for response and survival (PP population; Figure 1). In one of these two patients treatment was stopped prematurely due to a severe adverse event (intestinal perforation). The other patient excluded from PP analysis experienced a rapid disease progression accompanied by a severe decline of overall performance, requiring a discontinuation of imatinib after 5 days of treatment. In regard to the PP population, no objective responses were observed. One patient (6%) achieved a stabilisation of disease for 2.6 months, whereas the remaining 15 patients (94%) experienced a tumour progression after 2–8 weeks. After disease progression, under imatinib, five patients (31%) were treated with other therapeutic regimens. One of these latter patients achieved a complete remission after radical surgery, which is still ongoing (15+ months).

### Survival analysis

At the time of data analysis (July 2004), the median follow-up time was 18.9 months and a total number of 16 deaths had occurred, all of them due to melanoma disease. The ITT population revealed a median overall survival of 3.9 months, with a 95% confidence interval (CI) of 2.8–5.3 months; the median time to progression was 1.9 months (95% CI 1.2–2.1 months). With regard to the PP population, the median overall survival was 4.2 months (95% CI 3.2–5.5 months); the median time to progression was 1.9 months (95% CI 1.6–2.2 months). The two patients who are still alive are now surviving 19+ and 25+ months after onset of imatinib treatment, respectively. No obvious correlation could be observed between the tyrosine kinase expression pattern measured by immunohistochemistry and the overall survival time of the patients. However, it should be noticed that both of the patients who are still alive stained positive for all three kinases, CD117/c-kit, PDGF-R*α* and -R*β*, by immunohistochemistry.

## DISCUSSION

The present study did not reveal any objective efficacy of imatinib in advanced metastastic melanoma. The proof of concept failed to show that melanoma cells expressing relevant amounts of the receptor tyrosine kinases c-kit and PDGF-R can be inhibited in their proliferative activity by imatinib *in vitro* and *in vivo*. The immunohistochemical workup of the tumour material gained from 12 study patients prior to the onset of imatinib treatment revealed a heterogenous protein expression pattern of the target receptor kinases. Thus, the assumed direct impact of the receptor expression pattern on the efficacy of imatinib treatment should have been visible by differences in tumour response or survival between receptor-positive and receptor-negative patients. No such differences were observed in this study. Additionally, tumour cell lines were established from tumour specimens of four study patients. Characterisation of these cell lines by immunohistochemistry revealed that, from completely negative expression patterns of the three receptor tyrosine kinases to completely positive patterns, all kinds of combinations were represented. Despite these heterogenous expression profiles, all melanoma cell lines tested negative for an *in vitro* antiproliferative effect of imatinib. No correlation between the receptor expression profile and the *in vitro* growth inhibition by imatinib could be detected. These results are in line with recently published data about melanoma-bearing athymic nude mice treated with imatinib without efficacy, regardless of whether the melanoma cells expressed PDGF-R or c-kit (McGary *et al*, 2004).

Similar results were recently reported from a clinical trial investigating imatinib as first- or second-line treatment in small cell lung cancer (SCLC) (Johnson *et al*, 2003). In all, 19 patients were included into this study, with 14 of them confirmed to have SCLC. No objective responses could be observed. One patient experienced a stabilisation of his disease. Only 21% of the study patients showed a CD117/c-kit expression on the tumour specimens obtained. The authors therefore argued that their trial remained inconclusive and further investigations on larger patient cohorts should be done to verify or falsify an anticancer effect of imatinib in SCLC.

It might be argued as one possible explanation for the observed negative results of our study as well, that a positive target molecule expression pattern was not necessary for a patient's inclusion. The majority of clinical trials testing a targeted therapy require the proof of a positive expression of the target molecule on the patient's tumour cells prior to inclusion, like, for example, in clinical trials testing trastuzumab in breast cancer (Vogel *et al*, 2002; Burstein *et al*, 2003). However, we found that 11 out of 12 (92%) of the tumour biopsies tested were positive for at least one of the three target tyrosine kinases. Three out of 12 tumour biopsies (25%) were positive for all three kinases; five out of 12 (42%) were positive for two of them; and three out of 12 (25%) were positive for one. Only one biopsy specimen (8%) was completely negative for all the three target molecules. Thus, the frequency and pattern of expression of the target molecules can be rated as sufficient for an investigation of the assumed antitumoral activity of imatinib.

Recent studies investigating the tyrosine kinase inhibitor gefitinib in non-small-cell lung cancer (NSCLC) showed that the outcome of patients treated with targeted anticancer agents does not necessarily correlate with the expression quality and quantity of its target molecule, in this case epidermal growth factor receptor (EGFR) (Parra *et al*, 2004). A recent study in colorectal cancer patients revealed clinical efficacy of the EGFR inhibitory antibody cetuximab in patients with immunohistochemical EGFR-negative tumours (Chung *et al*, 2005). Moreover, it could be shown that gefitinib in NSCLC exerts clinical efficacy in 10% of patients only, and this efficacy was associated with specific activating mutations in the *EGFR* gene (Lynch *et al*, 2004). In the light of these findings, the number of 16 patients evaluable for tumour response and survival in the present study might be too small to detect a comparably low response rate.

Despite the high clinical efficacy of imatinib in CML and GIST, it became evident that subgroups of patients do not respond to the drug, or relapse after an initial response, respectively. The clinical resistance to imatinib in CML is caused by two major mechanisms: an amplification of the *Bcr–Abl* gene or mutations in the catalytic domain of the protein (Gorre *et al*, 2001; Hochhaus *et al*, 2002; Roumiantsev *et al*, 2002; Azam *et al*, 2003). In GIST, mutations of kit or PDGF-R are made responsible for the observed resistance to imatinib (Heinrich *et al*, 2003; Chen *et al*, 2004). Trying to exclude mutations of the target receptor proteins in our study, we sequenced the genes of c-kit, PDGF-R*α* and -R*β* in the melanoma cell lines derived from the four study patients. We did not find any gene mutations that would lead to a change in the amino-acid sequence of the target molecules of interest. Nevertheless, gene sequencing could be carried out in cell lines derived from four patients only. Thus, we have no information about the mutation status of the other 14 patients.

Imatinib is known as a highly efficient drug with low toxicities (Druker *et al*, 2001). Thus, it seems noteworthy that we observed an unexpectedly high rate of side effects in our present study. Five out of 18 (28%) patients experienced CTC grade 3 or 4 toxicities, one of them with a fatal outcome. Four patients developed strong cutaneous adverse events, two of them CTC grade 3, that were published previously (Ugurel *et al*, 2003). Dose modifications became necessary in seven out of 18 patients (39%); in three out of 18 patients (17%) treatment had to be stopped completely. One possible reason for this increased rate of side effects might be the high dose of imatinib (800 mg d^−1^) used in this study. The common dose range used in CML is 400–600 mg day^−1^. Since melanoma is a solid tumour like GIST, the imatinib regimen of the present study was chosen according to the dose that was used in GIST at the time the present trial was designed, which was 400–1000 mg day^−1^ (van Oosterom *et al*, 2001). Another possible explanation for the high frequency of severe toxicities is the fact that the patient population enrolled into our study according to the inclusion criteria had extremely poor prognostic criteria with high tumour burden, poor performance status and high numbers of pretreatment regimens.

In conclusion, this first clinical trial of imatinib in melanoma did not reveal an efficacy of imatinib as rescue treatment of advanced metastatic patients. The expression profile of the target molecules of imatinib on tumour samples obtained from the study patients showed no impact on tumour response or overall survival. Severe toxicities occurred more frequently than expected, leading to dose modifications in 39% of the patients. However, assumed that imatinib might be effective in a smaller subgroup of melanoma patients only, the number of 16 patients evaluable for response and survival in the present study is too small for a negative proof of concept.

## Figures and Tables

**Table 1:** Primer sequences for amplification of PDGF-R*α* and -R*β*

**Primer pair**	**Oligonucleotide sequence (5′–3′)**	**Position^a^**
*PDGF-Rα 1*		
Forward	ATC TCC TTG GAG CTG AGA ACC G	1653–1674
Reverse	AAG ACC CGA CCA AGC ACT AGT C	1935–1914
		
*PDGF-Rα 2*		
Forward	CGA TGC AGC TGC CTT ATG ACT C	1869–1890
Reverse	ACA TAG CTC CGT GTG CTT TCA TC	2301–2279
		
*PDGF-Rα 3*		
Forward	AAG TCA GGC CCC ATT TAC ATC A	2135–2156
Reverse	GTC CCA GGA GGA CGT TGC	2621–2604
		
*PDGF-Rα 4*		
Forward	AGA TGA TAA CTC AGA AGG CCT TAC	2488–2511
Reverse	TCA CTG GTA GCG TGG TCA GGC	2910–2890
		
*PDGF-Rα 5*		
Forward	CGG CAT GAT GGT GGA TTC TAC T	2830–2851
Reverse	CTC TTC AGA GGT CTG CGA GCT G	3280–3259
		
*PDGF-Rα 6*		
Forward	GTA CCT TTC TGC CCG TGA AG	2700–2719
Reverse	GGA AGG AAC CCC TCG AAT CC	3434–3415
		
*PDGF-Rβ 1*		
Forward	ACG CAG GAG GTC ATC GTG GTG	1908–1928
Reverse	TTG TTG CGG TGC AGG TAG TC	2446–2427
		
*PDGF-Rβ 2*		
Forward	ATT CTC AGG CCA CGA TGA A	2227–2245
Reverse	GGG CAG AGG GAA CGT AGT TAT	2703–2683
		
*PDGF-Rβ 3*		
Forward	CCA ACT ACA TGG CCC CTT ACG	2662–2682
Reverse	TTT TGT AAC CTT CGC CCA ACA	3258–3238
		
*PDGF-Rβ 4*		
Forward	GAC TGT TGG GCG AAG GTT ACA	3235–3255
Reverse	CAG TGG GCC CTC GTC AGC	3470–3453
		
*PDGF-Rβ 5*		
Forward	GGG TTC CAT GGC CTC CGA TCT	3336–3356
Reverse	GTT TGG GGC ACA ACA CGT CAG	3832–3812

a Nucleotide positions are shown according to the PDGF-R*α* and -R*β* coding sequences, accession NM_006206 and NM_002609, respectively.

**Table 2:** Patient characteristics

*ITT population*	18	(100.0%)
Sex		
Male	11	(61%)
Female	7	(39%)
		
Median age (years)	54.2 (Range 38.9–72.0)	
		
LDH		
⩽UNL	5	(28%)
>UNL	13	(72%)
		
Performance status (ECOG)		
0	6	(33%)
1	9	(50%)
2	3	(17%)
		
Metastatic sites^a^		
Skin/lymph nodes	18	(100%)
Lung	11	(61%)
Liver	5	(28%)
Other visceral	6	(33%)
Bone	1	(6%)
		
M category (AJCC)		
M1a	1	(6%)
M1b	1	(6%)
M1c	16	(88%)
		
Pretreatment (in stage IV)^a^		
Chemotherapy	18	(100%)
Immunotherapy	11	(61%)
1 therapy regimen	8	(44%)
2 therapy regimens	8	(44%)
⩾3 therapy regimens	2	(12%)
PP population^b^		16

a Multiple entries possible.

b Reasons for exclusion from evaluation see study flow (Figure 1). ITT, intention to treat; LDH, lactate dehydrogenase; UNL, upper normal limit; ECOG, Eastern Cooperative Oncology Group; AJCC, American Joint Committee on Cancer; PP, per protocol.

**Table 3:** Immunohistochemical analysis

Tissue specimen	12	(100%)
Origin of specimen		
Subcutaneous met	8	(66%)
Lymph node met	2	(17%)
Liver met	2	(17%)
		
CD117/c-kit		
+++	1	(8%)
++	2	(17%)
+	6	(50%)
−	2	(17%)
ND	1	(8%)
		
PDGF-R*α*		
+++	0	(0%)
++	0	(0%)
+	7	(58%)
−	5	(42%)
		
PDGF-R*β*		
+++	1	(8%)
++	0	(0%)
+	7	(58%)
−	4	(34%)

Biopsies were obtained from metastatic lesions of 12 study patients prior to the onset of imatinib therapy. Processing of tissue specimens, see Patients and Methods. The staining intensity of melanoma cells was graded as follows: +++, strong; ++, moderate; +, weak; −, negative. Met, metastasis; ND, not done.

**Table 4:** Tyrosine kinase expression pattern in melanoma tissues and corresponding cell lines

	**Patient #02**	**Patient #05**	**Patient #16**	**Patient #18**
	**Female**	**Male**	**Female**	**Male**
	**51 years**	**62 years**	**41 years**	**52 years**
*Tissue*				
Origin	Subcutaneous	Subcutaneous	Lymph node	Lymph node
Immunohistochemistry				
CD117/c-kit	++	+++	+	++
PDGF-R*α*	+	−	+	+
PDGF-R*β*	+	+	−	−
				
*Cell line*				
Name	Ma-Mel-46	Ma-Mel-52	Ma-Mel-54a	Ma-Mel-59a
Immunohistochemistry				
CD117/c-kit	+	−	++	−
PDGF-R*α*	−	−	++	++
PDGF-R*β*	++	−	+	−
				
RT–PCR				
CD117/c-kit	+	+	+	(+)
PDGF-R*α*	+	+	+	+
PDGF-R*β*	+	+	+	+
				
*Patient's clinical outcome*				
Overall survival (days)	88	7	20	74

Expression of tyrosine kinases was measured in melanoma tissue samples and corresponding cell lines as described in Patients and Methods. The patients are numbered according to the time point of inclusion into the study.

**Table 5:** Treatment-related toxicity and dose modification

**Sex (*years of age*)**	**CTC^a^ grade 1–2**	**CTC grade 3**	**CTC grade 4**	**Dose modification (%)^b^**
m (42)	Headaches, nausea, fatigue			−25
				
m (71)	Nausea, gum bleeding			−25
				
f (41)		Exanthema		−50
f (51)		Exanthema		−50
m (62)		Constipation	Intestinal perforation	−100
				
m (67)			Arterial thromboembolism	−100
				
m (61)			Suicide attempt	−100

a Toxicity was graded according to common toxicity criteria (CTC; http://ctep.cancer.gov/reporti ng/ctc.html).

b Modification of imatinib dose in terms of reduction of the initial dose of 400 mg bid. A dose reduction of 100% represents complete cessation of study treatment.

## References

[bib1] Azam M, Latek RR, Daley GQ (2003) Mechanisms of autoinhibition and STI-571/imatinib resistance revealed by mutagenesis of BCR–ABL. Cell 112: 831–843 12654249 10.1016/s0092-8674(03)00190-9

[bib2] Balch CM, Buzaid AC, Soong SJ, Atkins MB, Cascinelli N, Coit DG, Fleming ID, Gershenwald JE, Houghton Jr A, Kirkwood JM, McMasters KM, Mihm MF, Morton DL, Reintgen DS, Ross MI, Sober A, Thompson JA, Thompson JF (2001) Final version of the American Joint Committee on Cancer staging system for cutaneous melanoma. J Clin Oncol 19: 3635–3648 11504745 10.1200/JCO.2001.19.16.3635

[bib3] Burstein HJ, Harris LN, Marcom PK, Lambert-Falls R, Havlin K, Overmoyer B, Friedlander Jr RJ, Gargiulo J, Strenger R, Vogel CL, Ryan PD, Ellis MJ, Nunes RA, Bunnell CA, Campos SM, Hallor M, Gelman R, Winer EP (2003) Trastuzumab and vinorelbine as first-line therapy for HER2-overexpressing metastatic breast cancer: multicenter phase II trial with clinical outcomes, analysis of serum tumor markers as predictive factors, and cardiac surveillance algorithm. J Clin Oncol 21: 2889–2895 12885806 10.1200/JCO.2003.02.018

[bib4] Chen LL, Trent JC, Wu EF, Fuller GN, Ramdas L, Zhang W, Raymond AK, Prieto VG, Oyedeji CO, Hunt KK, Pollock RE, Feig BW, Hayes KJ, Choi H, Macapinlac HA, Hittelman W, Velasco MA, Patel S, Burgess MA, Benjamin RS, Frazier ML (2004) A missense mutation in KIT kinase domain 1 correlates with imatinib resistance in gastrointestinal stromal tumors. Cancer Res 64: 5913–5919 15342366 10.1158/0008-5472.CAN-04-0085

[bib5] Chung KY, Shia J, Kemeny NE, Shah M, Schwartz GK, Tse A, Hamilton A, Pan D, Schrag D, Schwartz L, Klimstra DS, Fridman D, Kelsen DP, Saltz LB (2005) Cetuximab shows activity in colorectal cancer patients with tumors that do not express the epidermal growth factor receptor by immunohistochemistry. J Clin Oncol 27, [epub ahead of print] 10.1200/JCO.2005.08.037 15677699

[bib6] Collisson EA, De A, Suzuki H, Gambhir SS, Kolodney MS (2003) Treatment of metastatic melanoma with an orally available inhibitor of the Ras-Raf-MAPK cascade. Cancer Res 63: 5669–5673 14522881

[bib7] Crosby T, Fish R, Coles B, Mason MD (2001) Systemic treatments for metastatic cutaneous melanoma (Cochrane Review). In: The Cochrane Library Vol 4 Oxford: Update Software

[bib8] Cross NC, Feng L, Bungey J, Goldman JM (1993) Minimal residual disease after bone marrow transplant for chronic myeloid leukaemia detected by the polymerase chain reaction. Leuk Lymphoma 11: 39–43 8251914 10.3109/10428199309047861

[bib9] Deininger MW, Goldman JM, Lydon N, Melo JV (1997) The tyrosine kinase inhibitor CGP57148B selectively inhibits the growth of BCR–ABL-positive cells. Blood 90: 3691–3698 9345054

[bib10] Demetri GD, von Mehren M, Blanke CD, Van den Abbeele AD, Eisenberg B, Roberts PJ, Heinrich MC, Tuveson DA, Singer S, Janicek M, Fletcher JA, Silverman SG, Silberman SL, Capdeville R, Kiese B, Peng B, Dimitrijevic S, Druker BJ, Corless C, Fletcher CD, Joensuu H (2002) Efficacy and safety of imatinib mesylate in advanced gastrointestinal stromal tumors. N Engl J Med 347: 472–480 12181401 10.1056/NEJMoa020461

[bib11] DiPaola RS, Kuczynski WI, Onodera K, Ratajczak MZ, Hijiya N, Moore J, Gewirtz AM (1997) Evidence for a functional kit receptor in melanoma, breast, and lung carcinoma cells. Cancer Gene Ther 4: 176–182 9171936

[bib12] Druker BJ, Talpaz M, Resta DJ, Peng B, Buchdunger E, Ford JM, Lydon NB, Kantarjian H, Capdeville R, Ohno-Jones S, Sawyers CL (2001) Efficacy and safety of a specific inhibitor of the BCR–ABL tyrosine kinase in chronic myeloid leukemia. N Engl J Med 344: 1031–1037 11287972 10.1056/NEJM200104053441401

[bib13] Eigentler TK, Caroli UM, Radny P, Garbe C (2003) Palliative therapy of disseminated malignant melanoma: a systematic review of 41 randomised clinical trials. Lancet Oncol 4: 748–759 14662431 10.1016/s1470-2045(03)01280-4

[bib14] Gorre ME, Mohammed M, Ellwood K, Hsu N, Paquette R, Rao PN, Sawyers CL (2001) Clinical resistance to STI-571 cancer therapy caused by BCR–ABL gene mutation or amplification. Science 293: 876–880 11423618 10.1126/science.1062538

[bib15] Harsh GR, Keating MT, Escobedo JA, Williams LT (1990) Platelet derived growth factor (PDGF) autocrine components in human tumor cell lines. J Neurooncol 8: 1–12 2156959 10.1007/BF00182081

[bib16] Heinrich MC, Corless CL, Demetri GD, Blanke CD, von Mehren M, Joensuu H, McGreevey LS, Chen CJ, Van den Abbeele AD, Druker BJ, Kiese B, Eisenberg B, Roberts PJ, Singer S, Fletcher CD, Silberman S, Dimitrijevic S, Fletcher JA (2003) Kinase mutations and imatinib response in patients with metastatic gastrointestinal stromal tumor. J Clin Oncol 21: 4342–4349 14645423 10.1200/JCO.2003.04.190

[bib17] Hochhaus A, Kreil S, Corbin AS, La Rosee P, Muller MC, Lahaye T, Hanfstein B, Schoch C, Cross NC, Berger U, Gschaidmeier H, Druker BJ, Hehlmann R (2002) Molecular and chromosomal mechanisms of resistance to imatinib (STI571) therapy. Leukemia 16: 2190–2196 12399961 10.1038/sj.leu.2402741

[bib18] Jansen B, Wacheck V, Heere-Ress E, Schlagbauer-Wadl H, Hoeller C, Lucas T, Hoermann M, Hollenstein U, Wolff K, Pehamberger H (2000) Chemosensitisation of malignant melanoma by BCL2 antisense therapy. Lancet 356: 1728–1733 11095261 10.1016/S0140-6736(00)03207-4

[bib19] Johnson BE, Fischer T, Fischer B, Dunlop D, Rischin D, Silberman S, Kowalski MO, Sayles D, Dimitrijevic S, Fletcher C, Hornick J, Salgia R, Le Chevalier T (2003) Phase II study of imatinib in patients with small cell lung cancer. Clin Cancer Res 9: 5880–5887 14676110

[bib20] Longley Jr BJ, Metcalfe DD, Tharp M, Wang X, Tyrrell L, Lu SZ, Heitjan D, Ma Y (1999) Activating and dominant inactivating c-KIT catalytic domain mutations in distinct clinical forms of human mastocytosis. Proc Natl Acad Sci USA 96: 1609–1614 9990072 10.1073/pnas.96.4.1609 PMC15534

[bib21] Lynch TJ, Bell DW, Sordella R, Gurubhagavatula S, Okimoto RA, Brannigan BW, Harris PL, Haserlat SM, Supko JG, Haluska FG, Louis DN, Christiani DC, Settleman J, Haber DA (2004) Activating mutations in the epidermal growth factor receptor underlying responsiveness of non-small-cell lung cancer to gefitinib. N Engl J Med 350: 2129–2139 15118073 10.1056/NEJMoa040938

[bib22] McGary EC, Onn A, Mills L, Heimberger A, Eton O, Thomas GW, Shtivelband M, Bar-Eli M (2004) Imatinib mesylate inhibits platelet-derived growth factor receptor phosphorylation of melanoma cells but does not affect tumorigenicity *in vivo*. J Invest Dermatol 122: 400–405 15009722 10.1046/j.0022-202X.2004.22231.x

[bib23] Montone KT, van Belle P, Elenitsas R, Elder DE (1997) Proto-oncogene c-kit expression in malignant melanoma: protein loss with tumor progression. Mod Pathol 10: 939–944 9310959

[bib24] Mosmann T (1983) Rapid colorimetric assay for cellular growth and survival: application to proliferation and cytotoxicity assays. J Immunol Methods 65: 55–63 6606682 10.1016/0022-1759(83)90303-4

[bib25] Parra HS, Cavina R, Latteri F, Zucali PA, Campagnoli E, Morenghi E, Grimaldi GC, Roncalli M, Santoro A (2004) Analysis of epidermal growth factor receptor expression as a predictive factor for response to gefitinib (‘Iressa’, ZD1839) in non-small-cell lung cancer. Br J Cancer 91: 208–212 15187994 10.1038/sj.bjc.6601923 PMC2409824

[bib26] Peng B, Hayes M, Resta D, Racine-Poon A, Druker BJ, Talpaz M, Sawyers CL, Rosamilia M, Ford J, Lloyd P, Capdeville R (2004) Pharmacokinetics and pharmacodynamics of imatinib in a phase I trial with chronic myeloid leukemia patients. J Clin Oncol 22: 935–942 14990650 10.1200/JCO.2004.03.050

[bib27] Potti A, Hille RC, Koch M (2003) Immunohistochemical determination of HER-2/neu overexpression in malignant melanoma reveals no prognostic value, while c-Kit (CD117) overexpression exhibits potential therapeutic implications. J Carcinogen 2: 8 10.1186/1477-3163-2-8 PMC280698 14617373

[bib28] Redondo P, Lloret P, Andreu EJ, Inoges S (2004) Imatinib mesylate in cutaneous melanoma. J Invest Dermatol 123: 1208–1209 15610538 10.1111/j.0022-202X.2004.23496.x

[bib29] Roumiantsev S, Shah NP, Gorre ME, Nicoll J, Brasher BB, Sawyers CL, Van Etten RA (2002) Clinical resistance to the kinase inhibitor STI-571 in chronic myeloid leukemia by mutation of Tyr-253 in the Abl kinase domain P-loop. Proc Natl Acad Sci USA 99: 10700–10705 12149456 10.1073/pnas.162140299 PMC125018

[bib30] Shen SS, Zhang PS, Eton O, Prieto VG (2003) Analysis of protein tyrosine kinase expression in melanocytic lesions by tissue array. J Cutan Pathol 30: 539–547 14507401 10.1034/j.1600-0560.2003.00090.x

[bib31] Sumimoto H, Miyagishi M, Miyoshi H, Yamagata S, Shimizu A, Taira K, Kawakami Y (2004) Inhibition of growth and invasive ability of melanoma by inactivation of mutated BRAF with lentivirus-mediated RNA interference. Oncogene June 21, [epub ahead of print] 10.1038/sj.onc.1207812 15208655

[bib32] Therasse P, Arbuck SG, Eisenhauer EA, Wanders J, Kaplan RS, Rubinstein L, Verweij J, Van Glabbeke M, van Oosterom AT, Christian MC, Gwyther SG (2000) New guidelines to evaluate the response to treatment in solid tumors. European Organization for Research and Treatment of Cancer, National Cancer Institute of the United States, National Cancer Institute of Canada. J Natl Cancer Inst 92: 205–216 10655437 10.1093/jnci/92.3.205

[bib33] Ugurel S, Hildenbrand R, Dippel E, Hochhaus A, Schadendorf D (2003) Dose-dependent severe cutaneous reactions to imatinib. Br J Cancer 88: 1157–1159 12698177 10.1038/sj.bjc.6600893 PMC2747559

[bib34] Ugurel S, Schadendorf D (2003) Systemic treatment in advanced melanoma: innovative perspectives. Onkologie 26: 234–238 12845207 10.1159/000071618

[bib35] van Oosterom AT, Judson I, Verweij J, Stroobants S, Donato di Paola E, Dimitrijevic S, Martens M, Webb A, Sciot R, Van Glabbeke M, Silberman S, Nielsen OS (2001) Safety and efficacy of imatinib (STI571) in metastatic gastrointestinal stromal tumours: a phase I study. Lancet 358: 1421–1423 11705489 10.1016/s0140-6736(01)06535-7

[bib36] Vogel CL, Cobleigh MA, Tripathy D, Gutheil JC, Harris LN, Fehrenbacher L, Slamon DJ, Murphy M, Novotny WF, Burchmore M, Shak S, Stewart SJ, Press M (2002) Efficacy and safety of trastuzumab as a single agent in first-line treatment of HER2-overexpressing metastatic breast cancer. J Clin Oncol 20: 719–726 11821453 10.1200/JCO.2002.20.3.719

[bib37] Welker P, Schadendorf D, Artuc M, Grabbe J, Henz BM (2000) Expression of SCF splice variants in human melanocytes and melanoma cell lines: potential prognostic implications. Br J Cancer 82: 1453–1458 10780526 10.1054/bjoc.1999.1076 PMC2363371

[bib38] Worm M, Reichert U, Dippel E, Czarnetzki BM, Schadendorf D (1993) Expression of growth factor receptors on human melanoma cells: comparison of modulating effects of interferons and retinoids. Exp Dermatol 2: 217–223 7512881 10.1111/j.1600-0625.1993.tb00036.x

